# Purifying Buildings by Ozone

**Published:** 1911-04-15

**Authors:** 


### PURIFYING BUILDINGS BY OZONE.

A few months ago The Hospital described the arrangement for purifying the air of the Free Library at Chicago by means of ozonised air. A somewhat similar apparatus is in use at a well-known drapery emporium in the West End of London. The apparatus appears to follow very closely the lines that were suggested in an article in The Hospital a few years back. It was then pointed out that the fatigue which surgeons and nurses feel when standing a long time in the necessarily warm atmosphere of an operating theatre can be overcome by adding a supply of ozone, or oxygen, to the ordinary ventilating air current, controlled from the theatre. An ozonising apparatus, or a cylinder of compressed oxygen, can be arranged to discharge into the air duct leading to the operating theatre; a valve worked electrically from the operating theatre controls the admission of ozone or oxygen into the duct.

In the drapery premises now to be described there is an air duct with an air washer, on very much the same lines as the Plenum System. The air enters from the street and is washed on its way to the duct; a fan on the inside of the washer draws the air in, and forces it into the ducts leading to the different rooms. Outside the duct is the ozoniser. It is an electrical condenser, consisting of metal plates, which are alternately charged and discharged at a pressure of 1,000 volts from an alternating current transformer fixed in the neighbourhood; a fan forces filtered air between the plates of the condenser. After being ozonised the air is delivered by a pipe into the main air duct, between the air washer and the fan. In passing through the fan it is mixed with the washed air, and passes out into the different rooms. The distinguishing odour of ozone is quite plainly perceptible in every room where the ozonised air is delivered.

In addition to the above, in a number of the rooms, particularly those in which the workpeople are engaged, auxiliary ozonair apparatus are fixed at intervals along the ceiling; each apparatus consists of a fan and an electrical condenser, which takes its current from a transformer in the neighbourhood. Ozonised air is delivered by the auxiliary ozonisers to the rooms in which they are fixed. The vitiated air is carried off by main shafts leading to the top of the building, and exhaust fans are fixed to assist their exit.

